# Occurrence of *bla*_CTX-M-1,_ *qnr*B1 and virulence genes in avian ESBL-producing *Escherichia coli* isolates from Tunisia

**DOI:** 10.3389/fcimb.2015.00038

**Published:** 2015-05-05

**Authors:** Hajer Kilani, Mohamed Salah Abbassi, Sana Ferjani, Riadh Mansouri, Senda Sghaier, Rakia Ben Salem, Imen Jaouani, Gtari Douja, Sana Brahim, Salah Hammami, Noureddine Ben Chehida, Ilhem Boutiba-Ben Boubaker

**Affiliations:** ^1^Laboratory of Bacteriological Research, Institut de la Recherche Vétérinaire de Tunis, Université de Tunis El ManarTunis, Tunisia; ^2^LR99ES09 Laboratoire de Résistance aux Antimicrobiens, Faculté de Médecine de Tunis, Université de Tunis El ManarTunis, Tunisia; ^3^Hôpital Charles Nicolle, Service de MicrobiologieTunis, Tunisia; ^4^Regional Animal Health Center for North Africa (RAHC-NA)Tunis, Tunisia; ^5^École Nationale de Médecine Vétérinaire de Sidi ThabetSidi Thabet, Tunisia

**Keywords:** *Escherichia coli*, poultry, *bla*_CTX-M-1_, *qnr*B1, integrons, clonality

## Abstract

Avian ESBL-producing *Escherichia coli* isolates have been increasingly reported worldwide. Animal to human dissemination, via food chain or direct contact, of these resistant bacteria has been reported. In Tunisia, little is known about avian ESBL- producing *E*. *coli* and further studies are needed. Seventeen ESBL-producing *Escherichia coli* isolates from poultry feces from two farms (Farm 1 and farm 2) in the North of Tunisia have been used in this study. Eleven of these isolates (from farm 1) have the same resistance profile to nalidixic acid, sulfonamides, streptomycin, tetracycline, and norfloxacine (intermediately resistant). Out of the six isolates recovered from farm 2, only one was co-resistant to tetracycline. All isolates, except one, harbored *bla*_CTX-M-1_ gene, and one strain co-harbored the *bla*_TEM-1_ gene. The genes *tetA* and *tetB* were carried, respectively, by 11 and 1 amongst the 12 tetracycline-resistant isolates. Sulfonamides resistance was encoded by *sul1*, *sul2*, and *sul3* genes in 3, 17, and 5 isolates, respectively. The *qnrB1* was detected in nine strains, one of which co-harbored *qnrS1* gene. The search for the class 1 and 2 integrons by PCR showed that in farm 1, class 1 and 2 integrons were found in one and ten isolates, respectively. In farm 2, class 1 integron was found in only one isolate, class 2 was not detected. Only one gene cassette arrangement was demonstrated in the variable regions (VR) of the 10 *int2*-positive isolates: *dfrA1*- *sat2*-*aadA1*. The size of the VR of the class 1 integron was approximately 250 bp in one *int1*-positive isolate, whereas in the second isolate, no amplification was observed. All isolates of farm 1 belong to the phylogroup A (sub-group A0). However, different types of phylogroups in farm 2 were detected. Each of the phylogroups A1, B2_2_, B2_3_ was detected in one strain, while the D2 phylogroup was found in 3 isolates. The virulence genes *iut*A, *fimH*, and *traT* were detected in 3, 7, and 3 isolates, respectively. Two types of gene combination were detected: *iutA*+*fimH*+*traT* in 3 isolates and *iutA*+*fimH* in one isolate. The isolates recovered in farm 1 showed the same profile of PFGE macro-restriction, while isolates of farm 2 presented unrelated PFGE patterns. We conclude that these avian ESBL-producing *E*. *coli* isolates show homo- and heterogenic genetic background and that plasmids harboring ESBL genes could be involved in the dissemination of this resistance phenotype.

## Introduction

*Escherichia coli* is a commensal bacterium in humans and animals and considered an indicator of fecal contamination of food. Antimicrobial resistant isolates and resistance genes of *E. coli* can be transferred to humans through the food chain. This transfer represents a potential risk for public health (Alexander et al., 2010; Cortés et al., 2010; Canton et al., 2012; Ryu et al., 2012). The use of broad-spectrum cephalosporins in animals, such as ceftiofur and cefquinome, has been recognized a major driving force for the selection and spread of extended spectrum beta-lactamases (ESBL) (Dutil et al., 2010). In both reservoirs, high prevalence of genes belonging to CTX-M group dominates. Numerous data highlight the extent to which certain ESBL genes, ESBL plasmids or ESBL-producing clones are shared between animals and humans (Naseer and Sundsfjord, 2011).

In recent years, there has been increasing concern in the scientific community about the emergence and dissemination of *E. coli* strains producing ESBLs, especially of the CTX-M class, which are very frequently associated with community infections (Eckert et al., 2004; Livermore et al., 2007; Pitout and Laupland, 2008). Recently, different reports have indicated the dissemination of ESBL-positive *E. coli* strains among the intestinal microbiota of healthy humans (Vinué et al., 2009), in food producing animals, and in food products (Brinas et al., 2005; Blanc et al., 2006; Girlich et al., 2007; Jouini et al., 2007; Li et al., 2007; Smet et al., 2008). These resistant bacteria could be transferred to humans through the food chain. This transfer represents a problem for public health. Comparison of human and animal ESBL-producing isolates is important in enhancing the knowledge of the potential routes of transfer of these bacteria and resistance genes in different ecosystems. In Tunisia, ESBL-producers were initially reported from food samples such as raw chicken meat (Jouini et al., 2007; Ben Slama et al., 2010). More recently, ESBL-producing *E. coli* were described in healthy food animals at farm level (Ben Sallem et al., 2012b, 2013; Mnif et al., 2012). In Tunisia, ESBL-producing bacteria were found in chickens and a dromedary, suggesting that poultry constitutes a major reservoir of ESBL genes. The dominant ESBL gene found was *bla*_CTX−M−1_, which was mainly detected with *Inc*I1 replicons (Mnif et al., 2012). A recent study also demonstrated that the *bla*_CTX−M−1_ IncI1/ST3 plasmid was dominant in Tunisian chickens and pets (Grami et al., 2013). Finally, recent data showed that 7.3% of Tunisian healthy humans were fecal carriers of CTX-M-1-producing *E. coli*. This finding suggests that foodstuff of poultry origin may contribute to the transmission of the *bla*_CTX−M−1_ gene from animals to humans (Ben Sallem et al., 2012b).

The aim of this study was to characterize ESBL producing *E. coli* isolates recovered from feces of healthy chickens in Tunisia by investigating genes encoding ESBL, resistance to tetracycline, sulfonamides and fluoroquinolones as well as content of virulence genes, integrons, and genetic clonality using PFGE.

## Materials and methods

### Bacterial strains

Sixty-five fecal samples were collected from healthy chickens in 2013. Of these samples, 45 came from 58-week-old chickens in a farm located at Sidi Thabet in the North of Tunisia (Farm 1) and 20 from 7-week-old chickens in a farm located at Morneg region (Farm 2). These samples were cultivated on Mac-Conkey agar containing 2 mg/L of cefotaxime and incubated overnight at 37°C. For each sample, one colony with typical *E. coli* trait was picked and re-isolated on Mac-Conkey agar and the phenotypic identification result was confirmed using Api20E (Bio-Mérieux, France).

### Antimicrobial susceptibility testing and ESBL identification

Antimicrobial susceptibility testing was carried out using the agar disk diffusion method on Mueller–Hinton agar plates in accordance with the Clinical and Laboratory Standards Institute guidelines (Clinical and Laboratory Standards Institute, 2012). The following antimicrobial agents were tested (μg/disk): ampicillin (10), amoxicillin (25), amoxicillin-clavulanic acid (20/10), ceftazidime (30), cefotaxime (30), gentamicin (10), kanamycin (30), streptomycin (10), amikacin (30), trimethoprim-sulfamethoxazol (1.25/23.75), tetracycline (30), nalidixic acid (30), ciprofloxacin (5), sulfonamides (200) and chloramphenicol (50). The Double-Disk Synergy Test (DDST) with cefotaxime or ceftazidime in the proximity to amoxicillin-clavulanic acid was used for the screening of ESBL (Clinical and Laboratory Standards Institute, 2012). *E. coli* ATCC25922 was used as ESBL negative and *Klebsiella pneumoniae* 700603 was used as ESBL positive reference strain.

### Resistance genotype

All primers used to characterize the resistance genotype are presented in Table 1. The presence of genes encoding TEM, SHV, CTX-M implicated in the beta-lactam resistance was analyzed by PCR (Sáenz, et al., 2004; Batchelor et al., 2005). Amplified DNA fragments were sequenced on both strands and the nucleotide and their deduced amino acid sequences were compared with those included in the Gen-Bank database as well as with those deposited at the website http://www.lahey.org/Studies/ in order to confirm the specific type of β-lactamase gene (Sáenz, et al., 2004; Batchelor et al., 2005). Genes encoding resistance to tetracycline (*tetA*, *tetB*, and *tetC*), sulfonamide (*sul1*, *sul2*, and *sul3*), and quinolones (*qnrA*, *qnrB*, and *qnrS*) were investigated by PCR as reported previously (Sáenz, et al., 2004; Wang et al., 2008; Rocha-Gracia et al., 2010). For positive isolates, PCR products of *qnrB* and *qnrS* were sequenced. The presence and characterization of integrons were studied by PCR of the class 1 and 2 integrase encoding genes as well as the 3′ conserved region (*qacE*Δ1 + *sul1* genes) (Table 1) and by PCR and subsequent sequencing of the variables regions (VRs) of these integrons (Sáenz, et al., 2004).

**Table 1:** **Primers of genes encoding resistance genes and integrons used in PCR of resistance genotype**.

**Primer name**	**Sequence (5′-3′)**	**Target gene or region**	**PCR products (bp)**	**References**
intI1-F	GGGTCAAGGATCTGGATTTCG	*intI1*	483	Sáenz, et al., 2004
intI1-R	ACATGCGTGTAAATCATCGTCG			
Int-F	GGCATCCAAGCAGCAAG	Class 1 integron variable region	Variable	Sáenz, et al., 2004
Int-R	AAGCAGACTTGACCTGA			
intI2-F	CACGGATATGCGACAAAAAGGT	*intI2*	788	Sáenz, et al., 2004
intI2-R	GTAGCAAACGAGTGACGAAATG			
Hep-F	CGGGATCCCGGACGGCATGCACGATTTGTA	Class 2 integron variable region	Variable	Sáenz, et al., 2004
Hep-R	GATGCCATCGCAAGTACGAG			
Qac-F	GGCTGGCTTTTTCTTGTTATCG	*qacE*Δ1-*sul1* region	1125	Sáenz, et al., 2004
SUL1-R	GCGAGGGTTTCCGAGAAGGTG			
SUL1-F	TGGTGACGGTGTTCGGCATTC	*sul1*	789	Sáenz, et al., 2004
SUL1-R	GCGAGGGTTTCCGAGAAGGTG			
SUL2-F	CGGCATCGTCAACATAACC	*sul2*	722	Sáenz, et al., 2004
SUL2-R	GTGTGCGGATGAAGTCAG			
SUL3-F	CATTCTAGAAAACAGTCGTAGTTCG	*sul* 3	990	Sáenz, et al., 2004
SUL3-R	CATCTGCAGCTAACCTAGGGCTTTGGA			
TetA-F	GTAATTCTGAGCACTGTCGC	*tetA*	937	Sáenz, et al., 2004
TetA-R	CTGCCTGGACAACATTGCTT			
TetB-F	CTCAGTATTCCAAGCCTTTG	*tetB*	416	Sáenz, et al., 2004
TetB-R	CTAAGCACTTGTCTCCTGTT			
TetC-F	TCTAACAATGCGCTCATCGT	*tetC*	570	Sáenz, et al., 2004
TetC-R	GGTTGAAGGCTCTCAAGGGC			
aac(6')-Ib-F	TTGCGATGCTCTATGAGTGGCTA	*aac(6′)-Ib*	482	Rocha-Gracia et al., 2010
aac(6')-Ib-R	CTCGAATGCCTGGCGTGTTT			
QepA-F	GGACATCTACGGCTTCTTCG	*qepA*	671	Rocha-Gracia et al., 2010
QepA-R	CAACTGCTTGAGCCCGTAG			
QnrA-F	GGGTATGGATATTATTGATAAA	*qnrA*	580	Rocha-Gracia et al., 2010
QnrA-R	CTAATCCGGCAGCACTATTA			
QnrBnew-F	GATCGTGAAAGCCAGAAAGG	*qnrB*	468	Wang et al., 2008
QnrBnew-R	ACGATGCCTGGTAGTTGTCC			
QnrS-F	AGTGATCTCACCTTCACCGC	*qnrS*	550	Rocha-Gracia et al., 2010
QnrS-R	CAGGCTGCAATTTTGATACC			
TEM-F	ATTCTTGAAGACGAAAGGGC	*bla*TEM	1150	Sáenz, et al., 2004
TEM-R	ACGCTCAGTGGAACGAAAAC			
SHV-F	CACTCAAGGATGTATTGTG	*bla*SHV	885	Sáenz, et al., 2004
SHV-R	TTAGCGTTGCCAGTGCTCG			
CTXM-Univ-F	CGATGTGCAGTACCAGTAA	*bla*CTXM	585	Batchelor et al., 2005
CTXM-Univ-R	TTAGTGACCAGAATCAGCGG			

### Virulence genotyping

The presence of 30 virulence genes (*fimA, TartT, iutA, MaIX, Ibe, FyuA, BmaE, papGalleleIII, papC, colV, cdtB, papG alleleI, nfaE, SfaS, iha, iss, ire, ehxA, sxt1, sxt2, eltA, fasA, estII, aggC, esat1, cdt, ipah, hly, cnf1*, and *bfp*) was determined by using PCR in all ESBL-positive *E. coli* strains (Chapman et al., 2006; Wu et al., 2007).

### Clonal and phylogenetic analysis of *E. coli* isolates

Chromosomal DNA was prepared as previously described using the restriction enzyme *XbaI* (Amersham Life Sciences, Uppsala, Sweden) (Kaufmann, 1998). DNA fragments were separated by electrophoresis in 1.2% agarose gels (pulsed-field agarose certified; Bio-Rad, Hemel Hempstead, United Kingdom) and 0.5 X Tris-borate-EDTA buffer using a contour-clamped homogeneous electric field (CHEF-DRIII system; Bio-Rad) under the following electrophoresis conditions: 12°C at 6 V/cm for 27 h with pulse times ranging from 10 to 40 s. Clonal relationships were established following Tenover criteria (Tenover et al., 1995). *E. coli* Isolates were allotted to phylogenetic groups A, B1, B2, or D, using a triplex PCR assay targeting the *chuA*, *yjaA* genes and the DNA fragment TSPE4.C2 (Clermont et al., 2000). Strains were sub-grouped according to Escobar-Paramo et al. (2006): subgroup A0: *chuA*-, *yjaA*-, and TspE4.C2-; subgroup A1: *chuA*-, *yjaA*+, and TspE4.C2-; group B1: *chuA*-, *yjaA*+/−, and TspE4.C2+; subgroup B2_2_: *chuA*+, *yjaA*+, and TspE4.C2-; subgroup B2_3_: *chuA*+, *yjaA*+, and TspE4.C2+; subgroup D1: *chuA*+, *yjaA*-, and TspE4.C2-; subgroup D2: *chuA*+, *yjaA*, and TspE4.C2+. Appropriate positive and negative controls were included in the assay.

## Results

### Occurrence of ESBL-producing *E. coli* isolates and antibiotic susceptibility

Eleven (24%) and 6 (30%) ESBL-producing *E. coli* isolates were detected in the 45 and 20 fecal samples collected in farm 1 and in farm 2, respectively. In addition to ESBL production, isolates in farm 1 have the same profile of resistance to nalidixic acid, norfloxacine (intermediate), trimethoprim-sulfamethoxazole, sulfonamides, streptomycin and tetracycline. But, they remained susceptible to imipenem, gentamicin, tobramycin and chloramphenicol. With the exception of one strain that was co-resistant to tetracycline, isolates, from farm 2, were susceptible to all non beta-lactam antibiotics (Table 2).

**Table 2:** **Phenotypic and molecular characteristics of the 17 ESBL-producing *E. coli* isolates**.

***E. coli* strain**	Resistance profil^*^	**Phylogenetic group**	**β-Lactamase (s)**	Int^**^	VR^**^ (bp)	***tet***	***sul***	PMQR^**^	**Virulence factors**	**PFGE type**
Ec139	NA, Nor^I^, Sxt, S, Tet	Ao	CTX-M-1	2	2000	A	*sul 2*	*qnrB1*	*iut A*	A
Ec149	NA, Nor^I^, Sxt, S, Tet	Ao	CTX-M-1	2	2000	A	*sul 1-2*	-	*iut A*	A
Ec174	NA, Nor^I^, Sxt, S, Tet	Ao	CTX-M-1	2	2000	A	*sul 2*	-	*iut A*	A
Ec143	NA, Nor^I^, Sxt, S, Tet	Ao	CTX-M-1	2	2000	A	*sul 2*	*qnrB1*	*iut A*	A
Ec147	NA, Nor^I^, Sxt, S, Tet	Ao	CTX-M1	2	2000	A	*sul 2*	-	*iut A*	A
Ec154	NA, Nor^I^, Sxt, S, Tet	Ao	CTX-M1	2	2000	A	*sul 2*	*qnrB1*	*iut A*	A
Ec146	NA, Nor^I^, Sxt, S, Tet	Ao	CTX-M1	2	2000	A	*sul 1-2*	-	*iut A*	A
Ec172	NA, Nor^I^, Sxt, S, Tet	Ao	CTX-M-1+ TEM-1	1	250	A	*sul 2-3*	*qnrB1*	*iut A, traT, fimH*	A
Ec156	NA, Nor^I^, Sxt, S, Tet	Ao	CTX-M-1	2	2000	A	*sul 2*	*qnrB1*, *qnrS1*	-	A
Ec151	NA, Nor^I^, Sxt, S, Tet	Ao	CTX-M-1	2	2000	A	*sul 2*	*qnrB1*	*iut A*	A
Ec173	NA, Nor^I^, Sxt, S, Tet	Ao	CTX-M-1	2	2000	A	*sul 2*	*qnrB1*	*iut A*	A
Ec67	-	A1	CTX-M-1	1	-	-	*sul 1-2*	-	*fimH*	B
Ec68	TET	B2_2_	-	-	-	B	*sul 2-3*	-	*iut A, traT, fimH*	C
Ec69	-	D_2_	CTX-M-1	-	-	-	*sul 2*	*qnrB1*	*iut A*, *traT*, *fimH*	D
Ec71	-	B2_3_	CTX-M-1	-	-	-	*sul 2-3*	*qnrB1*	*iut A*, *fimH*	E
Ec 72	-	D_2_	CTX-M-1	-	-	-	*sul 2-3*	-	*fimH*	F
Ec 76	-	D_2_	CTX-M-1	-	-	-	*sul 2-3*	-	*fimH*	G

* *Resistance to other antibiotic in addition to ESBL production, NA, nalidixic acid; Nor, Norfloxacine; Sxt, trimethoprim-sulfamethozole; S, streptomycin; Tet, tetracycline*.

** *Int, integron class; VR, variable region; PMQR, Plasmid Mediated Quinolone Resistance*.

### Gene coding for the production of ESBL

PCR and sequencing showed that all strains, except one, harbored the *bla*_CTX−M−1_ genes. One strain co-harbored *bla*_TEM−1_ gene. The *bla*_SHV_ gene was not detected.

### Occurrence of class 1 and 2 integrons

In farm 1, class 1 and 2 integrons were found in one and ten isolates, respectively. In farm 2, class 1 integron was found in only one isolate, while class 2 was not detected. Amplification of the VRs of class 2 integrons showed identical DNA fragments with an approximate size of 2000 bp. All these VRs contained a unique gene cassette arrangement, being *dfrA1*- *sat2*-*aadA1*, encoding resistance for trimethoprim, streptothricin and streptomycin, respectively. In one isolate, the VR of class 1 integron was amplified and yielded a DNA fragment of ca. 250 bp, whereas no amplification was observed in the second *int1*-positive isolate.

### Determination of phylogroups and virulence factors

In farm 1, all isolates were found to belong to phylogroup A (sub-group A0); however, different phylogroups were detected in farm 2 (A1, B2_2,_ and B2_3_, each in one isolate, and D_2,_ in 3 isolates). The virulence genes *iut(A)*, *fimH*, and *traT* were detected in 3, 7, and 3 isolates, respectively. The other genes were not detected in our collection. Two types of gene combination were detected: *iutA+fimH+traT* (in 3 isolates); *iutA+fimH* (in one isolate).

### Genes encoding tetracycline-, sulphonamide, and fluroquinolones resistance

Amongst the 12 tetracycline-resistant isolates, eleven and one carried *tetA* and *tetB*, respectively. Sulfonamides resistance was encoded by *sul1*, *sul2* and *sul3* genes in 3, 17 and 5 isolates, respectively. Gene *qnrB1* was detected in 9 isolates, one of them co-harbored *qnrS1* gene.

### PFGE typing

**Figure 1:**
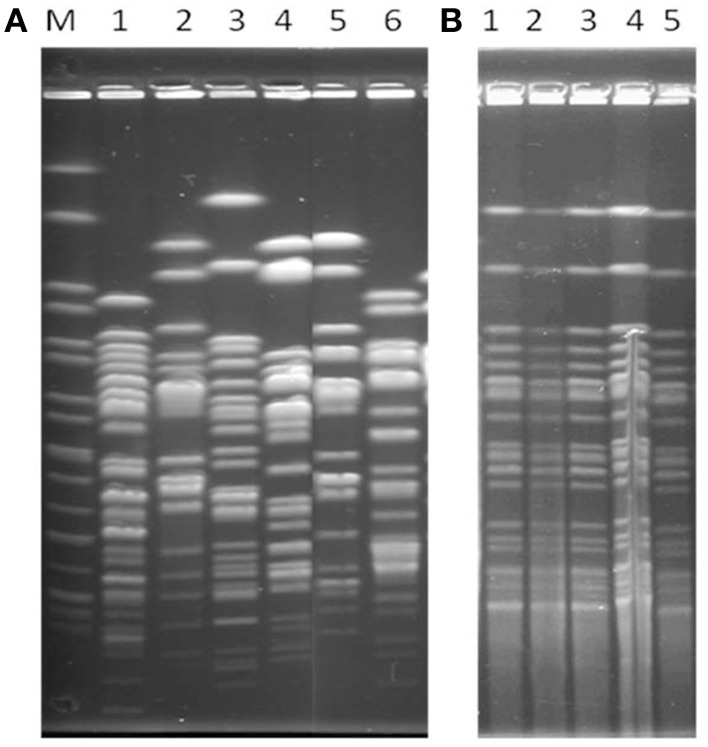
***Xba*I-PFGE profiles of representative *E. coli* isolates**. (**A**, Unrelated isolates) Lanes M, *Xba*I-digested DNA of *Salmonella enterica* serovar Braenderup H9812, used as size standard; Lanes 1–6, Ec 67, Ec68, Ec69, Ec71, Ec72; (**B**, Clonal isolates) Lanes 1 to5, EC139, EC149, Ec 174, Ec143, Ec147, respectively.

All isolates from farm 1 were clonally related, while isolates of the farm 2 were genetically unrelated (Figure 1).

## Discussion

We collected 65 samples of feces from 45 reproductive 58-weeks-old chickens in a public farm in the region of Sidi Thabet (30 Km Nord-West of Tunis, Tunisia) (Farm1) and 20 samples of 7-weeks-old breeder chickens in the region of Morneg (Farm 2). Seventeen samples (26.1%) contained cefotaxime-resistant isolates, 11 (24%) and 6 (30%) positives samples were observed in farms 1 and 2, respectively. The frequency of cefotaxime-resistant isolates is similar to those reported by other authors worldwide (2%; 31.7%) (Dierikx et al., 2013; Randall et al., 2014), but lower than those found by other studies in Tunisia (42% and 45, 5%) (Ben Sallem et al., 2012b; Mnif et al., 2012). In our study, all isolates from farm 1 exhibited the same resistance profile to nalidixic acid, trimethoprim-sulfamethoxazole, sulfonamides, streptomycin, tetracycline and norfloxacine (intermediately resistant). These resistance markers are often reported in previous Tunisian studies (Ben Slama et al., 2010; Mnif et al., 2012). All isolates were susceptible to imipenem, gentamicin, kanamycin and chloramphenicol. Indeed, low rates of resistance to these antibiotics have been reported worldwide (Grami et al., 2013). However, the 6 isolates from farm 2, except one that was co-resistant to tetracycline, were susceptible to all antibiotics tested. Worldwide, the CTX-M group has been emerged as the predominant determinant encoding ESBL production in human and animal *Enterobacteriaceae*, especially *E. coli* (Girlich et al., 2007; Coque et al., 2008; Nicolas-Chanoine et al., 2008; Dahmen et al., 2012, 2013). The *bla*_CTX−M−1_ gene was amplified in 16 out of 17 isolates; in addition, one isolate co-harbored the *bla*_TEM−1_ gene. This result is in agreement with those of other Tunisian findings (Jouini et al., 2007, 2009; Ben Slama et al., 2010; Ben Sallem et al., 2012b, 2013; Mnif et al., 2012). The high rate occurrence of *E. coli* harboring *bla*_CTX−M−1_ in poultry and other food products of animal origin can contribute to the transmission of this gene or these strains to humans. Indeed, it was recently demonstrated that *E. coli* containing CTX-M-1 was identified in 7.3% of healthy Tunisians (Ben Sallem et al., 2012a). Identical or closely related isolates from humans and animals have been previously reported in Netherlands, suggesting a likely transmission of ESBL-*E. coli* isolates from poultry to human, most probably via the food chain (Overdevest et al., 2011). More recently, Chinese data showed a high occurrence of CTX-M-14 in isolates from animals where this enzyme is very widespread in human isolates (Zheng et al., 2012). In the EC68 strain, no *bla* gene was detected. This strain could harbor a gene not investigated in our study. Further investigation must be undertaken. Antibiotic resistance, especially the multiresistance, has been mainly linked to the dissemination of linked genes encoding resistance inserted in mobile genetic elements, mainly integrons. Integron of class 1 was found in two isolates while integron of class 2 in ten isolates. Our results are not in agreement with other findings, which showed the dominance of integron of class 1 in animal-derived *E. coli* or in animal products as well as human isolates (Machado et al., 2005; Soufi et al., 2009, 2011; Ben Slama et al., 2010; Cergole-Novella et al., 2010). Two hypotheses could explain our finding: firstly, the real absence of class 1 integrons in the integron-free isolates, as reported by other authors (Jouini et al., 2007; Ben Sallem et al., 2012b). Secondly, the presence of insertion sequences, such as *IS*26, truncating the *intI* and thus, leading to inhibition of PCR amplification. In the case of truncated *intI* gene by *IS*26, the use of other primers was proposed (Marchant et al., 2013). Owing to a number of financial limitations, we could not realize these PCRs actually. The then *int2*-positive isolates presented an identical gene cassette array in their VRs: *dfrA*-*sat2*-*aadA1*, being frequent in other studies (Soufi et al., 2009, 2011). The integrase Int2 is not functional and thus unable to integrate new gene cassettes into the variable region, other than those already present (Partridge et al., 2009). The variable region of the class 2 integron mainly carries *dfrA1* (encoding trimethoprim resistance), *sat1* (encoding streptothricin resistance) and *aadA1* (encoding streptomycin/spectinomycin resistance) (Partridge et al., 2009; Soufi et al., 2009, 2011). The size of the VR of class 1 integron in EC172 was approximately 250 bp, a fragment that cannot correspond to any gene cassette of the known ones (Ravi et al., 2014). The VR of class 1 integron in EC67 was not amplified; this might suggest an empty VR or mismatches of used primers.

Tetracycline resistance was encoded by *tetA* and *tetB* genes in 11 and one tetracycline-resistant isolates, respectively. This finding was also reported by other studies (Koo and Woo, 2011; Xibiao et al., 2011), while the *tetC* gene is rarely reported (Skoèková et al., 2013). The *sul1*, *sul2* and *sul3* genes were detected in 3, 17, and 5 isolates, respectively, not only amongst sulfonamid resistant isolates, but also in the six-sulfonamide susceptible isolates. In other reports, *sul1* is most frequently reported followed by *sul2* gene while *sul3* gene is generally less so (Sköld, 2000; Perreten and Boerlin, 2003; Hammerum et al., 2006; Trobos et al., 2008). Resistance toward quinolone and fluoroquinolones is mainly due to target mutations in quinolone resistance determining region (QRDR) of DNA gyrase (*gyrA* and *gyrB*) and topoisomerases IV (*parC* and *parE*) (Hawkey, 2003). However, Plasmid Mediate Quinolone Resistance (PMQR) genes such as *qnrA*, *qnrB*, *qnrC*, *qnrD*, *qnrS*, *qepA*, and *aac(6′)Ib-cr* have been increasingly reported in bacterial pathogens since 2000 (Strahilevitz et al., 2009). In our study, among 11 isolates resistant to nalidixic acid and intermediately susceptible sensibility to norfloxacine, seven isolates carried *qnrB1* gene and one also co-heberged *qnrS1* gene. These two genes were often reported by other authors worldwide (Ben Sallem et al., 2013, 2014; Ferjani et al., 2014) in *E. coli* producers of CTX-M from human and animal origin. In our study, we did not consider *qepA* and *aac (6′)-Ib-cr* genes, while other authors reported their presence in *E. coli* from animal origin (Ma et al., 2009; Xie et al., 2014). The *qnr* gene types have no big effect on the increase of minimal inhibitory concentration (MIC) of fluoroquinolones (ciprofloxacine and norfloxacine) and were generally not detected by disk diffusion method. Indeed, the resistance toward quinolones in 7 amongst the 11 nalidixic acid resistant isolates could be explained by these *qnr* genes but it could also certainly explained by the presence of chromosomal mutations in QRDR of GyrA and/or ParC (Abbassi et al., 2010). The quinolone-susceptible isolate containing *qnrB1* gene confirmed the low level expression of this resistance (in term of increase of MIC).

Concerning the distribution of pathogenic *E. coli* strains according to phylogroups, it is well-known that pathogenic strains producing extra-intestinal infections (ExPEC) belong mainly to the B2 group and less to the D group. They are responsible for meningitidis, abscess, peritonitis, septicemia and urinary tract infections (Le Gall et al., 2007; Nandanwar et al., 2014); while, groups A and B1 *E. coli* strains are considered non-virulent commensal strains (Ewers et al., 2007). All the strains of farm 1 were found to belong to the phylogroup A (sub-type A0), while, the strains of farm 2 to phylogroups A (A1, 1 isolate), D (D2, 3 isolates) and B2 (B2_2_, 1 isolate; B2_3_, 1 isolate). In the literature, the majority of animal *E. coli* isolates producing ESBL belonged to phylogroup A and B1 (Mnif et al., 2012; Huber et al., 2013) contrary to human isolates, which mainly belonged to B2 phylogroup. The low number of virulence genes detected was in relation with the appurtenance of our isolates (12/17) to the phylogroup A. Strains of phylogroups B2 and D were somewhat distinguished from strains of phylogroup A by the occurrence of the fimbrial *fimH* gene and the serum resistance-associated outer membrane (*traT*). The low number of genes detected could be explained by fitness notion or the competitively of strains. Indeed, these avian intestinal *E. coli* isolates were under selective presser by antibiotics. This selective pressure enhances the expansion and maintenance of antibiotic-resistant strains rather than virulent ones.

PFGE showed that the 11 isolates of the farm 1 were indistinguishable, while isolates of farm 2 were unrelated. The cloanlity of these isolates would evoke a strong power of dissemination of clonal isolates in this poultry breeding. The difference in genetic contents in isolates of farm2 (*sul* genes, *qnr* genes and virulence genes) might be due to independent acquisition of genes carried by plasmids or integrons.

## Conclusion

Despite the limited samples analyzed in this study, that might not reflect the real epidemiological situation of avian ESBL-producing *E*. *coli* in these two farms, our study showed two typical epidemiologic characteristics of ESBL-producing bacteria. Firstly, clonal isolates are disseminated within the same farm. Secondly, “singleton” isolates occur with limited ability of spread, evoking the potential horizontal transfer of ESBL genes between different *E. coli* populations. Plasmids or integrons might be implicated in the mobilization of *bla*_CTX−M−1_ genes among avian isolates. Further studies should be performed in the future to track the evolution of ESBL types and their frequencies in different ecosystems.

## Author contributions

HK, MA, RM, SH, NC, and IB: Conceiving and designing the study, collecting and interpreting the data, and writing the article. SS, RS, IJ, and SF: interpreting the data, revising the article.

### Conflict of interest statement

The authors declare that the research was conducted in the absence of any commercial or financial relationships that could be construed as a potential conflict of interest.

## References

[B1] Abbassi M. S. Ruiz E. Saenz Y. Mechergui A. Ben Hassen A. Torres C. (2010). Genetic background of quinolones resistance in CTX-M-15-producing *Klebsiella pneumonia* and *Escherichia coli* strains in Tunisia. J. Chemother. 22, 66–67. 10.1179/joc.2010.22.1.66 20227997

[B2] Alexander T. W. Inglis G. D. Yanke L. J. Topp E. Read R. R. Reuter T.. (2010). Farm-to-fork characterization of *Escherichia coli* associated with feedlot cattle with a known history of antimicrobial use. Int. J. Food Microbiol. 137, 40–48. 10.1016/j.ijfoodmicro.2009.11.008 19963297

[B2a] Batchelor M. Hopkins K. Threlfall E. J. Clifton-Hadley F. A. Stallwood A. D. Davies R. H.. (2005). *bla*_CTX-M_ genes in clinical *Salmonella* isolates recovered from humans in England and Wales from 1992 to 2003. Antimicrob. Agents Chemother. 49, 1319–1322. 10.1128/AAC.49.4.1319-1322.2005 15793104 PMC1068621

[B3] Ben Sallem R. Ben Slama K. Estepa V. Jouini A. Gharsa H. Klibi N.. (2012a). Prevalence and characterization of extended-spectrum beta-lactamase (ESBL)-producing *Escherichia coli* isolates in healthy volunteers in Tunisia. Eur. J. Clin. Microbiol. Infect. Dis. 31, 1511–1516. 10.1007/s10096-011-1471-z 22065280

[B4] Ben Sallem R. Ben Slama K. Sáenz Y. Rojo-Bezares B. Estepa V. Jouini A.. (2012b). Prevalence and characterization of extended-spectrum beta-lactamase (ESBL)- and CMY-2-producing *Escherichia coli* isolates from healthy food-producing animals in Tunisia. Foodborne Pathog. 9, 1137–1142. 10.1089/fpd.2012.1267 23194332

[B5] Ben Sallem R. Gharsa H. Ben Slama K. Rojo-Bezares B. Estepa V. Porres-Osante N. (2013). First detection of CTX-M-1, CMY-2, and QnrB19 resistance mechanisms in fecal *Escherichia coli* isolates from healthy pets in Tunisia. Vector Borne Zoonotic Dis. 13, 98–102. 10.1089/vbz.2012.1047 23289399

[B5a] Ben Sallem R. Ben Slama K. Rojo-Bezares B. Porres-Osante N. Jouini A. Klibi N.. (2014). IncI1 plasmids carrying *bla*_CTX-M-1_ or *bla*_CMY-2_ genes in *Escherichia coli* from healthy humans and animals in Tunisia. Microb. Drug. Resist. 20, 495–500. 10.1089/mdr.2013.0224 24826863

[B6] Ben Slama K. Jouini A. Bem Sallem R. Somalo S. Saenz Y. Estepa V.. (2010). Prevalence of broad-spectrum cephalosporin-resistant *Escherichia coli* isolates in food samples in Tunisia, and characterization of integrons and antimicrobial resistance mechanisms implicated. Int. J. Microbiol. Food. 137, 281–286. 10.1016/j.ijfoodmicro.2009.12.003 20031243

[B7] Blanc V. Mesa R. Saco M. Lavilla S. Prats G. Miró E.. (2006). ESBL- and plasmidic class C beta-lactamase-producing *E. coli* strains isolated from poultry, pig and rabbit farms. Vet. Microbiol. 118, 299–304. 10.1016/j.vetmic.2006.08.002 16973308

[B8] Brinas L. Moreno M. A. Teshager T. Saenz Y. Porrero M. C. Dominguez L.. (2005). Monitoring and characterization of extended-spectrum beta-lactamases in *Escherichia coli* strains from healthy and sick animals in Spain in 2003. Antimicrob. Agents Chemother. 49, 1262–1264. 10.1128/AAC.49.3.1262-1264.2005 15728945 PMC549238

[B9] Canton R. Gonzales-Alba J. M. Galan R. C. (2012). CTX-M- enzymes: origin and diffusion. Front. Microbiol. 3:110. 10.3389/fmicb.2012.00110 22485109 PMC3316993

[B10] Cergole-Novella M. C. Guth B. E. Castanheira M. Carmo M. S. Pignatari A. C. (2010). First description of *bla*_CTX-M-14_- and *bla*_CTX-M-15_-producing *Escherichia coli* isolates in Brazil. Microb. Drug Resist. 16, 177–184. 10.1089/mdr.2010.0008 20704513

[B11] Chapman T. A. Wu X. Y. Barchia I. Bettelheim K. A. Driesen S. Trott D.. (2006). Comparison of virulence gene profiles of *Escherichia coli* strains isolated from healthy and diarrheic swine. Appl. Environ. Microbiol. 72, 4782–4795. 10.1128/AEM.02885-05 16820472 PMC1489375

[B12] Clermont O. Bonacorsi S. Bingen E. (2000). Rapid and simple determination of the *Escherichia coli* phylogenetic group. Appl. Environ. Microbiol. 66, 4555–4558. 10.1128/AEM.66.10.4555-4558.2000 11010916 PMC92342

[B13] Clinical and Laboratory Standards Institute. (2012). Performance Standards for Antimicrobial Susceptibility Testing; Twenty-second Informational Supplement. Clinical and Laboratory Standards Institute Document M100-S22. Wayne, PA: Clinical and Laboratory Standards Institute.

[B14] Coque T. M. Novais A. Carattoli A. Poirel L. Pitout J. Peixe L.. (2008). Dissemination of clonally related *Escherichia coli* strains expressing extendedspectrum beta-lactamase CTX-M-15. Emerg. Infect. Dis. 14, 195–200. 10.3201/eid1402.070350 18258110 PMC2600198

[B15] Cortés P. Blanc V. Mora A. Dahbi G. Blanco J. E. Blanco M.. (2010). Isolation and characterization of potentially pathogenic antimicrobial-resistant *Escherichia coli* strains from chicken and pig farms in Spain. Appl. Environ. Microbiol. 76, 2799–2805. 10.1128/AEM.02421-09 20228098 PMC2863447

[B16] Dahmen S. Haenni M. Madec J. Y. (2012). IncI1/ ST3 plasmids contribute to the dissemination of the *bla*_CTX-M-1_ gene in *Escherichia coli* from several animal species in France. J. Antimicrob. Chemother. 67, 3011–3012. 10.1093/jac/dks308 22872449

[B17] Dahmen S. Metayer V. Gay E. Madec J. Y. Haenni M. (2013). Characterization of extended-spectrum beta-lactamase (ESBL)-carrying plasmids and clones of Enterobacteriaceae causing cattle mastitis in France. Vet. Microbiol. 162, 793–799. 10.1016/j.vetmic.2012.10.015 23127568

[B18] Dierikx C. M. vander Goot J. A. Smith H. E. Kant A. Mevius D. J. (2013). Presence of ESBL/AmpC-producing *Escherichia coli* in the broiler production pyramid: a descriptive study. PLoS ONE 8:e79005. 10.1371/journal.pone.0079005 24244401 PMC3820706

[B19] Dutil L. R. Irwin R. Finley L. K. Ng B. Avery P. Boerlin A. M.. (2010). Ceftiofur resistance in *Salmonella enterica* serovar Heidelberg from chicken meat and humans, Canada. Emerg. Infect. Dis. 16, 48–54. 10.3201/eid1601.090729 20031042 PMC2874360

[B20] Eckert C. Gautier V. Saladin-Allard M. Hidri N. Verdet C. Ould-Hocine Z.. (2004). Dissemination of CTX-M-type β-lactamases among clinical isolates of *Enterobacteriaceae* in Paris, France. Antimicrob. Agents Chemother. 48, 1249–1255. 10.1128/AAC.48.4.1249-1255.2004 15047527 PMC375249

[B21] Escobar-Paramo P. Le Menac'h A. Le Gall T. Amorin C. Gouriou S. Picard B.. (2006). Identification of forces shaping the commensal *Escherichia coli* genetic structure by comparing animal and human isolates. Environ. Microbiol. 8, 1975–1984. 10.1111/j.1462-2920.2006.01077.x 17014496

[B22] Ewers C. Li G. Wilking H. Kiessling S. Alt K. Antáo E. M.. (2007). Avian pathogenic, uropathogenic, and newborn meningitis-causing *Escherichia coli*: how closely related are they? Int. J. Med. Microbiol. 297, 163–176. 10.1016/j.ijmm.2007.01.003 17374506

[B23] Ferjani S. Saidani M. Amine F. S. Boutiba-Ben Boubaker I. (2014). Prevalence and characterization of plasmid-mediated quinolone resistance genes in extended-spectrum β-lactamase-producing Enterobacteriaceae in a Tunisian hospital. Microb. Drug. Resist. 21, 158–166. 10.1089/mdr.2014.0053 25247633

[B24] Girlich D. Poirel L. Carattoli A. Kempf I. Lartigue M. F. Bertini A.. (2007). Extended-spectrum β-lactamase CTX-M-1 in *Escherichia coli* isolates from healthy poultry in France. Appl. Environ. Microbiol. 73, 4681–4685. 10.1128/AEM.02491-06 17513592 PMC1932829

[B25] Grami R. Mansour W. Dahmen S. Mehri W. Haenni M. Aouni M.. (2013). The *bla*_CTX-M-1_ IncI1/ST3 plasmids dominate in chickens and pets in Tunisia. J. Antimicrob. Chemother. 68, 2950–2952. 10.1093/jac/dkt258 23800900

[B26] Hammerum A. M Sandvang D. Andersen S. R. Seyfarth A. M. Porsbo L. J. Frimodt-Møller N.. (2006). Detection of *sul1*, *sul2* and *sul3* in sulphonamide resistant *Escherichia coli* isolates obtained from healthy humans, pork and pigs in Denmark. Int. J. Food Microbiol. 106, 235–237. 10.1016/j.ijfoodmicro.2005.06.023 16216373

[B27] Hawkey P. M. (2003). Mechanisms of quinolone action and microbial response. J. Antimicrob. Chemother. 51, 29–35. 10.1093/jac/dkg207 12702701

[B28] Huber H. Zweilfel C. Wittenbrink M. M. Stephan R. (2013). ESBL-Producing uropathogenic *Escherichia coli* isolated from dogs and cats in Switzerland. Vet. Microbiol. 162, 992–996. 10.1016/j.vetmic.2012.10.029 23177909

[B29] Jouini A. Ben Slama K. Saenz Y. Klibi N. Costa D. Vinué L.. (2009). Detection of multiple-antimicrobial resistance and characterization of the implicated genes in *Escherichia coli* isolates from foods of animal origin in Tunis. Food Prot. 72, 76–82. 19517738 10.4315/0362-028x-72.5.1082

[B30] Jouini A. Vinué L. Ben Slama K. Saenz Y. Klibi N. Hammami S.. (2007). Characterization of CTX-M and SHV extended-spectrum beta-lactamases and associated resistance genes in *Escherichia coli* strains of food samples in Tunisia. J. Antimicrob. Chemother. 60, 1137–1141. 10.1093/jac/dkm316 17855726

[B31] Kaufmann M. E. (1998). Pulsed-field gel electrophoresis, in Methods in Molecular Medicine, Vol. 15, Molecular Bacteriology: Protocols and Clinical Applications, eds Woodford N. Johnson A. P. (Totowa, NJ: Humana Press, Inc.), 17–31.

[B32] Koo H. J. Woo G. J. (2011). Distribution and transferability of tetracycline resistance determinants in *Escherichia coli* isolated from meat and meat products. Int. J. Food Microbiol. 145, 407–413. 10.1016/j.ijfoodmicro.2011.01.003 21324543

[B32a] Le Gall T. Clermont O. Gouriou S. Picard B. Nassif X. Denamur E.. (2007). Extraintestinal virulence is a coincidental by-product of commensalism in B2 phylogenetic group *Escherichia coli* strains. Mol. Biol. Evol. 24, 2373–2384. 10.1093/molbev/msm172 17709333

[B33] Li X. Z. Mehrotra M. Ghimire S. Adewoye L. (2007). Beta-lactam resistance and beta-lactamases in bacteria of animal origin. Vet. Microb. 121, 873–880. 10.1016/j.vetmic.2007.01.015 17306475

[B34] Livermore D. M. Canton R. Gniadkowski M. Nordmann P. Rossolini G. M. Arlet G.. (2007). CTX-M: changing the face of ESBLs in Europe. J. Antimicrob. Chemother. 59, 165–174. 10.1093/jac/dkl483 17158117

[B35] Ma J. Zeng Z. Chen Z. Xu X. Wang X. Deng Y.. (2009). High prevalence of plasmid-mediated quinolone resistance in determinants qnr, aac(6')-Ib-cr, and qepA among ceftiofur-resistant Enterobacteraceae isolates from companion and food-producing animals. Antimicrob. Agents Chemother. 53, 519–524. 10.1128/AAC.00886-08 18936192 PMC2630616

[B36] Machado E. Canton R. Baquero F. Galan J. C Rollan A. Peixe L.. (2005). Integron content of extended spectrum-β-lactamase-producing *Escherichia coli* strains over 12 years in a single hospital in Madrid, Spain. Antmicrob. Agents Chemother. 49, 1823–1829. 10.1128/AAC.49.5.1823-1829.2005 15855502 PMC1087637

[B37] Marchant M. Vinué L. Torres C. Moreno M. A. (2013). Change of integrons over time in *Escherichia coli* isolates recovered from healthy pigs and chickens. Vet. Microbiol. 163, 124–132. 10.1016/j.vetmic.2012.12.011 23290120

[B38] Mnif B. Ktari S. Rhimi F. M. Hammami A. (2012). Extensive dissemination of CTX-M-1-and CMY-2-producing *Escherichia coli* in poultry farms in Tunisia. Lett. Appl. Microbiol. 55, 407–413. 10.1111/j.1472-765X.2012.03309.x 22966763

[B39] Nandanwar N. Janssen T. Kühl M. Ahmed N. Ewers C. Wieler L. H. (2014). Extraintestinal pathogenic *Escherichia coli* (ExPEC) of human and avian origin belonging to sequence type complex 95 (STC95) portray indistinguishable virulence features. Int. J. Med. Microbiol. 304, 835–842. 10.1016/j.ijmm.2014.06.009 25037925

[B40] Naseer U. Sundsfjord A. (2011). The CTX-M conundrum: dissemination of plasmids and *Escherichia coli* clones. Microb. Drug. Resist. 17, 83–97. 10.1089/mdr.2010.0132 21281129

[B41] Nicolas-Chanoine M. H. Blanco J. Leflon-Guibout V. Demarty R. M. Alonso P. Caniça M. M.. (2008). Intercontinental emergence of *Escherichia coli* clones O25:H4-ST131 producing CTX-M-15. J. Antimicrob. Chemother. 61, 273–281. 10.1093/jac/dkm464 18077311

[B42] Overdevest I. Willemsen I. Rijnsburger M. Eustace A. Xu L. Hawkey P.. (2011). Extended-spectrum β-lactamase genes of *Escherichia coli* in chicken meat and humans, The Netherlands. Emerg. Infect. Dis. 17, 1216–1222. 10.3201/eid1707.110209 21762575 PMC3381403

[B43] Partridge S. R. Tsafnat G. Coiera E. Iredell J. R. (2009). Gene cassettes and cassette arrays in mobile resistance integrons. FEMS Microbiol. Rev. 3, 757–784. 10.1111/j.1574-6976.2009.00175.x 19416365

[B44] Perreten V. Boerlin P. (2003). A new sulfonamide resistance gene (*sul3*) in *Escherichia coli* is widespread in the pig population of Switzerland. Antimicrob. Agents. Chemother. 47, 1169–1172. 10.1128/AAC.47.3.1169-1172.2003 12604565 PMC149312

[B45] Pitout J. D. Laupland K. B. (2008). Extended-spectrum β-lactamase-producing *Enterobacteriaceae*: an emerging public-health concern. Lancet Infect. Dis. 8, 159–166. 10.1016/S1473-3099(08)70041-0 18291338

[B46] Randall L. Heinrich K. Horton R. Brunton L. Sharman M. Bailey-Horne V.. (2014). Detection of antibiotic residues and association of cefquinome residues with the occurrence of extended-spectrum β-lactamase (ESBL) producing bacteria in waste milk samples from dairy farms in England and Wales in 2011. Res. Vet. Sci. 96, 15–24. 10.1016/j.rvsc.2013.10.009 24314891

[B47] Ravi A. Avershina E. Ludvigsen J. L'Abée-Lund T. M. Rudi K. (2014). Integrons in the intestinal microbiota as reservoirs for transmission of antibiotic resistance genes. Pathogens 3, 238–248. 10.3390/pathogens3020238 25437798 PMC4243444

[B48] Rocha-Gracia R. Ruiz E. Romero-Romero S. Lozano-Zarain P. Somalo S. Palacios-Hernández J. M.. (2010). Detection of plasmid-borne quinolone resistance determinant *qepA1* in a CTX-M-15 producing *Escherichia coli* from Mexico. J Antimicrob. Chemother. 65, 169–171. 10.1093/jac/dkp418 19910326

[B49] Ryu S. H. Lee J. H. Park S. H. Song M. O. Park S. H. Jung H. W.. (2012). Antimicrobial resistance profiles among *Escherichia coli* strains isolated from commercial and cooked foods. Int. J. Food Microbiol. 159, 263–266. 10.1016/j.ijfoodmicro.2012.09.001 23107506

[B50] Sáenz Y. Briñas L. Dominguez E. Ruiz J. Zarazaga M. Vila J. (2004). Mechanisms of resistance in multiple-antibiotic-resistant *Escherichia coli* strains of human, animal, and food origins. Antimicrob. Agents Chemother. 48, 3996–4001 10.1128/AAC.48.10.3996-4001.2004 15388464 PMC521888

[B51] Skoèková A. Karpíšková R. Koláèková I. Cupáková Š. (2013). Characteristics of *Escherichia coli* from raw vegetables at a retail market in the Czech Republic. Int. J. Food Microbiol. 167, 196–201. 10.1016/j.ijfoodmicro.2013.09.011 24135675

[B52] Sköld O. (2000). Sulfonamide resistance: mechanisms and trends. Drug. Resist. Updat. 3, 155–160. 10.1054/drup.2000.0146 11498380

[B53] Smet A. Martel A. Persoons D. Dewulf J. Heyndrickx M. Catry B.. (2008). Diversity of extended-spectrum b-lactamases and class C beta-lactamases among cloacal *Escherichia coli* isolates in Belgian broiler farms. Antimicrob. Agents Chemother. 52, 1238–1243. 10.1128/AAC.01285-07 18227190 PMC2292556

[B54] Soufi L. Abbassi M. S. Saenz A. Vinué L. Somalo S. Zarazaga M.. (2009). Prevalence and diversity of integrons and associated resistance genes in *Escherichia coli* isolates from Poultry meat in Tunisia. Foodborne Patho. Dis. 6, 1067–1073. 10.1089/fpd.2009.0284 19642918

[B55] Soufi L. Sáenz Y. Vinué L. Abbassi M. S. Ruiz E. Zarazaga M.. (2011). *Escherichia coli* of poultry food origin as reservoir of sulphonamide resistance genes and integrons. Int. J. Food Microbiol. 144, 497–502. 10.1016/j.ijfoodmicro.2010.11.008 21131082

[B56] Strahilevitz J. Jacoby G. A. Hooper D. C. Robicsek A. (2009). Plasmid-mediated quinolone resistance: a multifaceted threat. Clin. Microbiol. Rev. 22, 664–689. 10.1128/CMR.00016-09 19822894 PMC2772364

[B57] Tenover F. C. Arbeit R. D. Goering R. V. Mickelsen P. A. Murray B. E. Persing D. H.. (1995). Interpreting chromosomal DNA restriction patterns produced by pulsed-field gel electrophoresis: criteria for bacterial strain typing. J. Clin. Microbiol. 33, 2233–2239. 7494007 10.1128/jcm.33.9.2233-2239.1995 PMC228385

[B58] Trobos M. Jakobsen L. Olsen K. E. Frimodt-Møller N. Hammerum A. M. Pedersen K.. (2008). Prevalence of sulphonamide resistance and class 1 integron genes in *Escherichia coli* isolates obtained from broilers, broiler meat, healthy humans and urinary infections in Denmark. Int. J. Antimicrob. Agents 32, 367–369. 10.1016/j.ijantimicag.2008.04.021 18583102

[B59] Vinué L. Saenz Y. Martinez S. Somalo S. Moreno M. A. Torres C.. (2009). Prevalence and diversity of extended spectrum beta-lactamases in faecal *Escherichia coli* isolates from healthy humans in Spain. Clin. Microbiol. Infect. 15, 954–957. 10.1111/j.1469-0691.2009.02803.x 19519849

[B60] Wang A. Yang Y. Lu Q. Wang Y. Chen Y Deng L. (2008). Presence of qnr gene in *Escherichia coli* and *Klebsiella pneumoniae* resistant to ciprofloxacin isolated from pediatric patients in China. BMC Infect. Dis. 22, 8–68. 10.1186/1471-2334-8-68 18498643 PMC2409344

[B61] Wu X. Y. Chapman T. Trott D. J. Bettelheim K. Do T. N. Driesen S.. (2007). Comparative analysis of virulence genes, genetic diversity and phylogeny between commensal and enterotoxigenic *Escherichia coli* from weaned pigs. Appl. Environ. Microbiol. 73, 83–91. 10.1128/AEM.00990-06 17056683 PMC1797122

[B62] Xibiao T. Chen T. Xuan Z. Zhanqin Z. Xin X. Bin W.. (2011). Antimicrobial resistances of extraintestinal pathogenic *Escherichia coli* isolates from swine in China. Microbial. Pathogenesis. 50, 207–212. 10.1016/j.micpath.2011.01.004 21237262

[B63] Xie R. Huo S. Li Y. Chen L. Zhang F. Wu X. (2014). Molecular epidemiological survey on quinolone resistance genotype and phenotype of *Escherichia coli* in septicemic broilers in Hebei, China. Poult. Sci. 93, 335–339. 10.3382/ps.2013-03522 24570454

[B64] Zheng H. Zeng Z. Chen S. Liu Y. Yao Q. Deng Y.. (2012). Prevalence and characterization of CTX-M β-lactamases amongst *Escherichia coli* isolates from healthy food animals in China. Int. J. Antimicrobiol. Agents 39, 305–310. 10.1016/j.ijantimicag.2011.12.001 22325120

